# *De novo* genome assembly and analysis of *Zalaria* sp. Him3, a novel fructooligosaccharides producing yeast

**DOI:** 10.1186/s12863-022-01094-2

**Published:** 2022-11-10

**Authors:** Jun Yoshikawa, Minenosuke Matsutani, Mayumi Maeda, Yutaka Kashiwagi, Kenji Maehashi

**Affiliations:** 1 Department of Fermentation Science, Faculty of Applied Bioscience, Tokyo University of Agriculture, 1-1-1 Sakuragaoka, Setagaya-ku, Tokyo, 156-8502 Japan; 2 NODAI Genome Research Center, Tokyo University of Agriculture, 1-1-1 Sakuragaoka, Setagaya-ku, Tokyo, 156-8502 Japan

**Keywords:** *Zalaria*, Genome assembly, Black yeast, β-fructofuranosidase

## Abstract

**Background:**

*Zalaria* sp. Him3 was reported as a novel fructooligosaccharides (FOS) producing yeast. However, *Zalaria* spp. have not been widely known and have been erroneously classified as a different black yeast, *Aureobasidium pullulans*. In this study, *de novo* genome assembly and analysis of *Zalaria* sp. Him3 was demonstrated to confirm the existence of a potential enzyme that facilitates FOS production and to compare with the genome of *A. pullulans*.

**Results:**

The genome of *Zalaria* sp. Him3 was analyzed; the total read bases and total number of reads were 6.38 Gbp and 42,452,134 reads, respectively. The assembled genome sequence was calculated to be 22.38 Mbp, with 207 contigs, N50 of 885,387, L50 of 10, GC content of 53.8%, and 7,496 genes. g2419, g3120, and g3700 among the predicted genes were annotated as cellulase, xylanase, and β-fructofuranosidase (FFase), respectively. When the read sequences were mapped to *A. pullulans* EXF-150 genome as a reference, a small amount of reads (3.89%) corresponded to the reference genome. Phylogenetic tree analysis, which was based on the conserved sequence set consisting of 2,362 orthologs in the genome, indicated genetic differences between *Zalaria* sp. Him3 and *Aureobasidium* spp.

**Conclusion:**

The differences between *Zalaria* and *Aureobasidium* spp. were evident at the genome level. g3700 identified in the *Zalaria* sp. Him3 likely does not encode a highly transfructosyl FFase because the motif sequences were unlike those in other FFases involved in FOS production. Therefore, strain Him3 may produce another FFase. Furthermore, several genes with promising functions were identified and might elicit further interest in *Zalaria* yeast.

**Supplementary Information:**

The online version contains supplementary material available at 10.1186/s12863-022-01094-2.

## Background

*Zalaria*, a black yeast, was isolated from various sources, such as house dust, blackened wooden artwork, and dried sweet potato in North America, Italy, and Japan, respectively [1–3]. Recently, *Zalaria* sp. Him3 was reported as a novel fructooligosaccharides (FOS) producer [3] and hence it is an attractive candidate for industrial production of FOS. However, it is not known what enzymes or substances this species produces besides FOS. Moreover, *Zalaria* strains were incorrectly classified as *Aureobasidium pullulans*, which is another species of black yeast in the same order Dothideales, and were required re-identification of *Zalaria* spp. [1]. This incorrect classification is also due to the fact that both species produce a melanin pigment when grown on agar media, which makes it difficult to distinguish them by their appearance alone [1, 3, 4].

*A. pullulans* has several applications in the biotechnological industry because the yeast produces various industrially important materials, such as pullulan, β-glucan, and FOS [5–7]. Pullulan and β-glucan are utilized for the production of oxygen-impermeable films and for its immunostimulant effects, respectively [5, 6, 8]. FOS, on the other hand, contributes to modulate the human gastrointestinal microbiota and is hence used as a prebiotic [9]. Additionally, some *A. pullulans* strains have been considered as biocontrol agents for crop protection to exhibit a strong inhibitory effect on plant pathogenic bacteria [10].

To the best of our knowledge, the genomes of most *Zalaria* spp. have not been analyzed unlike those of *A. pullulans* [4]. Furthermore, the available information on this species is limited because bioengineering studies using *Zalaria* have only focused on FOS production. Therefore, analysis of its genome would enhance our understanding of this yeast species and elucidate the expression of various enzymes and allow for comparison with other yeast species.

In the present study, *de novo* genome assembly and genome analysis of *Zalaria* sp. Him3 were demonstrated. Furthermore, its genome sequence was compared with that of *Aureobasidium* spp. as references to clarify the genetic differences between the two yeast species.

## Results

### *De novo* genome assembly of *Zalaria* sp. Him3

**Fig. 1:**
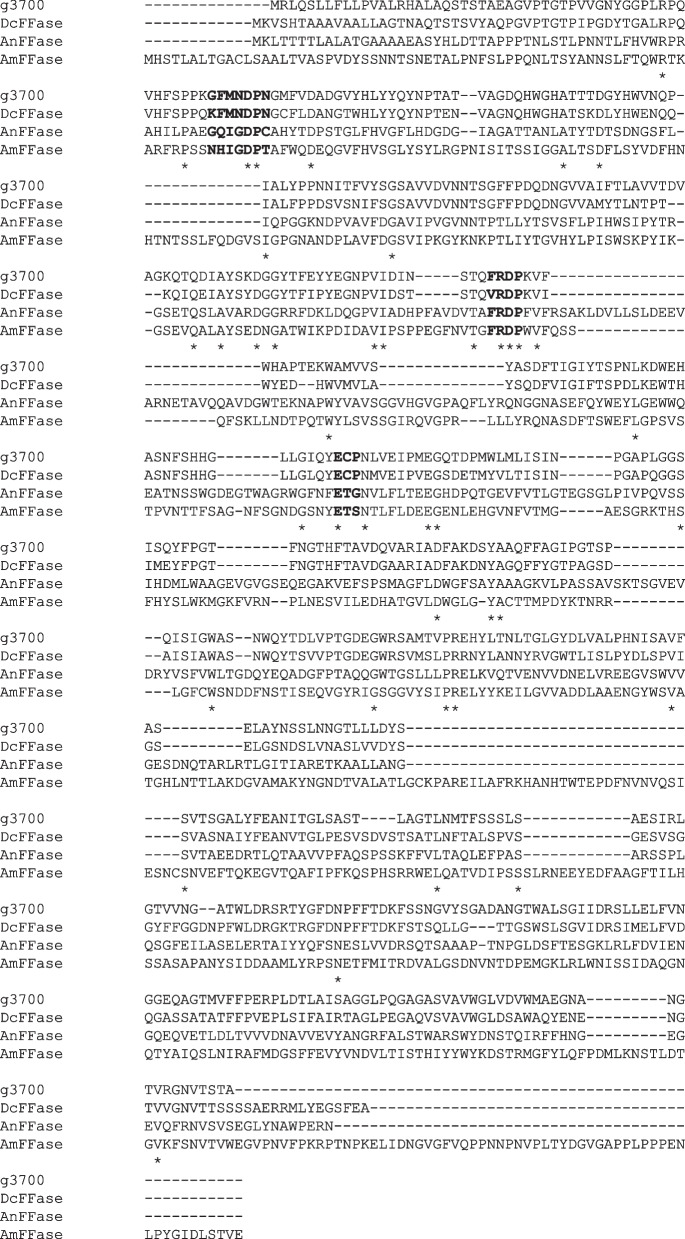
Multiple alignment with amino acids sequences of β-fructofuranosidase. The g3700 sequence was deduced from the transcript of *Zalaria* sp. Him3 genome. DcFFase, AmFFase, and AnFFase were β-fructofuranosidase in *Aureobasidium melanogenum* 11 − 1, *Aspergillus niger* ATCC 20611, and *Diplodia corticola* CBS 112549, respectively. The active sites predicted from AmFFase are indicated in bold. The conserved residues are indicated with an asterisk

The genome information of *Zalaria* spp. has not been investigated in detail. This is the first study to analyze the genome of *Zalaria* sp. Him3, a FOS producing yeast strain. The total bases and total number of reads in the raw data were 6.48 Gbp and 42,883,258 reads, respectively. The Q30 score, which is the ratio of bases that have a Phred quality score greater than 30, was 92.3%. The raw data were trimmed using Cutadapt [11], and the total base of 6.38 Gbp and the total read of 42,452,134 reads were obtained. FastQC analysis did not identify any issues with the sequence quality. The assembled genome sequence calculated using QUAST [12] was found to be 22.38 Mbp with 207 contigs, N50 of 885,387, L50 of 10, GC content of 53.8%, and 7,496 genes (Table 1). The genome coverage of the total sequenced bases (6.38 Gbp) was 285-fold of the genome size (22.38 Mbp). The quality assessment of the genome assembly was performed using BUSCO [13], and the completed BUSCO value in the data set of dothideomycetes_odb10 was 84.7% (3207 of 3786 genes). The predicted transcripts in the contigs (4022 genes) were annotated with BLAST search (Table S1). Among these transcripts, g3700 in contig NODE 9 was annotated as β-fructofuranosidase (FFase), which shared 73% sequence identity with that of *Diplodia corticola* CBS 112549 (DcFFase). Multiple alignments were constructed with the amino acid sequences of FFase from *Aureobasidium melanogenum* 11 − 1 (AmFFase) [14] and FFase from *Aspergillus niger* ATCC 20611 (AnFFase) [15], which are highly transfructosyl enzymes, in addition to DcFFase and the deduced amino acid sequence of g3700 (Fig. 1). These amino acid sequences were not highly conserved. Otherwise, g2419 and g3120 in the predicted transcripts were annotated as cellulase and xylanase, respectively, which are also carbohydrate degrading enzymes. Furthermore, gene clusters responsible for secondary metabolite production in the draft genome were identified by antiSMASH [16]. The regions from 255,015 to 301,675 in NODE 9 and 208,530 to 230,840 in NODE 16 corresponded with a melanin biosynthesis cluster in *Bipolaris oryzae* (Minimum Information about a Biosynthetic Gene cluster [MIBiG] accession: BGC0001265) and a clavaric acid biosynthesis cluster in *Hypholoma sublateritium* (MIBiG accession: BGC0001248), respectively.

**Table 1:** Statistics of *de novo* genome assembly of *Zalaria* sp. Him3

Statistics	Values
Reads^a^	42,452,134
Read bases (bp)^a^	6,383,865,743
Contigs^b^	207
Genome size (bp)^b^	22,376,659
N50^b^	885,387
L50^b^	10
GC (%)^b^	53.79
Predicted gene^c^	7,496

^a^ These values were calculated with Cutadapt ver. 2.10 [11]

^b^ These values were calculated with QUAST ver. 5.0.2 [12]

^c^ This value was calculated with AUGUSTUS ver. 3.3.3 [24]

### Comparison of *Zalaria* sp. Him3 genome sequence with *Aureobasidium* spp. genome as a reference

**Fig. 2:**
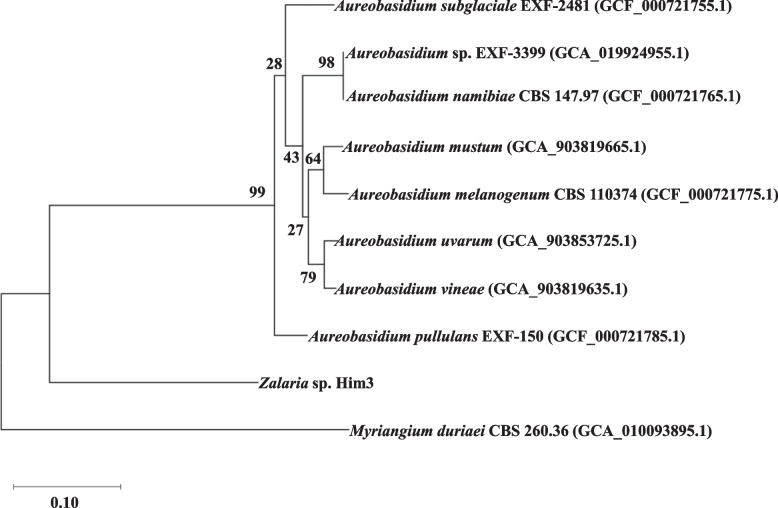
Phylogenetic tree analysis based on 2,362 orthologous sequences of *Zalaria* sp. Him3 and *Aurebasidium* spp. *M. duriaei* was used as an outgroup. Accession numbers are indicated in parentheses. Gene-support frequencies were calculated with reference to Salichos and Rokas [29]

An extensive comparison of orthologs between the genome of *Zalaria* and *Aureobasidium* has not been reported. Moreover, it is difficult to distinguish between *Zalaria* and *Aureobasidium* spp. based on their appearance alone because both are black yeasts. Only 3.89% reads from strain Him3 were mapped to the genome of *A. pullulans* EXF-150 [4], suggesting substantial divergence between the two genomes. The genome size (29.62 Mbp) of the strain EXF-150 was larger than that of the strain Him3 (22.34 Mbp). The GC contents of *Zalaria* sp. Him3 and *A. pullulans* EXF-150 were 53.8% and 50.0%, respectively. Phylogenetic tree analysis based on the concatenated sequence set consisting of 2,362 orthologs was performed for *Zalaria* sp. Him3, *Myriangium duriaei* CBS 260.36, and 8 strains of *Aureobasidium* spp. The average sequence identity for the 2362 orthologs was 81.0%. As shown in Fig. 2, the strain Him3 was found to be genetically distant from *Aureobasidium* spp. This result suggested that there were differences between the two yeast species at the genome level.

## Discussion

When sequences of the internal transcribed spacer region from strains of *Aureobasidium* and *Zalaria* spp. were compared by phylogenetic analysis, a portion of *Zalaria* strains was located in the *A. pullulans* clade [1, 3]. Humphries et al. reported that the strain ATCC 16628 was originally recognized as *A. pullulans* but was re-identified as *Zalaria obscura* [1]. The identification of *Zalaria* was insufficient because this yeast is a relatively new genus. An accurate classification of the *Zalaria* spp. is required to improve our understanding of this yeast species for future industrial applications. In the present study, genomic comparison revealed that *Zalaria* sp. Him3 has little genetic similarity with *Aureobasidium* spp. (Fig. 2), and this finding was also supported by the genome mapping rate. This result proved that there was a significant genetic difference between the two yeasts, *Zalaria* and *Aureobasidium*, and that the independency of the genus *Zalaria* was confirmed.

This is the first study to perform genome analysis of *Zalaria* sp. Him3. FFase gene (g3700) was identified from the predicted transcripts in the draft genome sequence. FFase is an important enzyme for the production of FOS [3]. *A. pullulans* DSM 2404 expresses multiple FFases for FOS production, and FFase I and IV showed high transfructosylating and hydrolytic activities, respectively [17]. Only g3700 was found in the Him3 genome, and this FFase gene did not exhibit high similarity with the high transfructosyl FFase, AmFFase and AnFFase (Fig. 1). The motifs (GQIGDP, RDP, and FET) for transfructosyl activity in GH32 FFase were previously reported in neighboring residues of the active sites [14, 18]. g3700 had the motifs for hydrolytic activity (WMNDPNGL, RDP, and ECP), although this enzymatic activity was not tested. Therefore, *Zalaria* sp. Him3 might express a different type of transfructosyl FFase, which might be important for FOS production. This yeast species might potentially play a role in biomass degradation [19] because g2419 and g3120 reportedly encode cellulase and xylanase, respectively. In terms of secondary metabolites, *Zalaria* spp. was suggested to possess the active gene cluster for melanin production because this yeast formed a melanotic colony when grown on agar media [1, 3]. Clavaric acid was reported to exert antitumor activity [20], and the related gene cluster was identified in the strain Him3, although that production has still not been confirmed. The present genome analysis may not be the best, but several promising genes were identified. This result could be expected to promote further analysis as a novel criterion for *Zalaria* yeast.

## Conclusion

In the present study, we performed *de novo* genome assembly of *Zalaria* sp. Him3. Phylogenetic analysis was performed for the concatenated 2,362 orthologous sequences, and the difference between *Aureobasidium* spp. and strain Him3 was evident. FFase gene (g3700) related to FOS production was annotated from the genome sequence, but the motif sequence suggested that the enzyme has a hydrolytic activity. This finding suggests that *Zalaria* sp. Him3 may produce a different type of FFase that facilitates FOS production. Additionally, genes related to carbohydrate degrading enzymes and secondary metabolites were also identified. These results extend the scope for further analysis of *Zalaria* spp. and highlight the potential of this yeast for various industrial applications.

## Methods

### Strain

*Zalaria* sp. Him3 strain was isolated from a Japanese dried sweet potato [3]. It was cultured on Yeast extract Peptone Dextrose (YPD) agar medium (2% glucose, 1% yeast extract, 2% polypeptone, and 1.5% agar) at 30 °C.

### Genome sequencing

*Zalaria* sp. Him3 strain, grown on YPD agar medium, was suspended in 10 mM Tris-HCl buffer (pH 8.0) containing 1 mM ethylenediaminetetraacetic acid, and the cell pellet was collected by centrifugation at 20,000 × *g* for 1 min. Genomic DNA was prepared using Dr. GenTLE (from Yeast) High Recovery Kit (Takara Bio Inc, Shiga, Japan). Approximately 1.5 µg of DNA was subjected to whole-genome sequencing. The DNA libraries were prepared using TruSeq DNA PCR-Free (Illumina, San Diego, CA, USA) according to the protocol. The prepared library was sequenced at 2 × 151 bp on NovaSeq 6000 (Illumina). Removal of the adapter sequences, sequences of less than 21 base reads, and other unwanted sequences, was performed for the sequenced paired-end reads using Cutadapt ver. 2.10 [11]. The trimmed data quality was validated with FastQC ver. 0.11.9 (Babraham Bioinformatics, Cambridge, UK; https://www.bioinformatics.babraham.ac.uk/projects/fastqc).

### Genome assembly and gene prediction

The trimmed data for *Zalaria* sp. Him3 genome was assembled using SPAdes ver. 3.14.1 [21] and mapped to the contigs with Burrows-Wheeler Aligner ver. 0.7.17 [22]. The contig sequences were improved for base differences and gaps with Pilon ver. 1.23 [23]. The genome assemble quality was validated with QUAST ver. 5.0.2 [12]. After coding sequences were identified from the contig sequences using AUGUSTUS ver. 3.3.3 [24] based on the *A. pullulans* genome sequence (txid1043002), the predicted transcripts were annotated using nucleotide BLAST with the NCBI Reference Sequence Database (RefSeq_rna). The coding sequences predicted using AUGUSTUS were evaluated with BUSCO ver. 4.1.3 [13], and the data set of dothideomycetes_odb10, orthologous genes from 45 species of the class Dothideomycetes in OrthoDB (https://www.orthodb.org), was used. Multiple alignments were constructed with translated sequences of g3700, DcFFase (accession number: XM_020274717), AmFFase (accession number: MH626577), and AnFFase (accession number: AB046383) using ClustalW program (https://www.genome.jp/tools-bin/clustalw). Gene clusters responsible for secondary metabolite production in the contig sequences were predicted using antiSMASH ver. 6.0.1 [16].

### Mapping of *Zalaria* sp. Him3 genome sequence to *A. pullulans* genome

The read data for *Zalaria* sp. Him3 were mapped to the *A. pullulans* EXF-150 genome (accession number: GCA_000721785.1) as a reference sequence using the Burrows-Wheeler Aligner ver. 0.7.17 [21]. The mapping rate was evaluated using Qualimap ver. 2.2.1 [25].

### Phylogenetic tree analysis

A phylogenetic tree based on the genome was constructed using RAxMLver. 8.2.2 [26]. The common 2,362 orthologous sequences were used for the analysis. Orthologous sets were identified from the genome sequences of *Zalaria* sp. Him3, *Aureobasidium meianogenum* CBS 110374 (accession number: GCF_000721775.1), *Aureobasidium mustum* (accession number: GCA_903819665.1), *Aureobasidium namibiae* CBS 147.97 (accession number: GCA_000721765.1), *A. pullulans* EXF-150 (accession number: GCF_000721785.0), *Aureobasidium subglaciale* EXF-2481 (accession number: GCF_000721755.1), *Aureobasidium uvarum* (accession number: GCA_903853725.1), *Aureobasidium vineae* (accession number: GCA_903819635.1), and *Aureobasidium* sp. EXF-3399 (accession number: GCA_019924955.1) using protein BLAST [27] as described by Matsutani et al. [28]. Furthermore, the orthologs were concatenated and analyzed after the alignment gaps of each sequence were removed. The gene-support frequency was calculated as described by Salichos and Rokas [29]. The sequence of *M. duriaei* CBS 260.36 (accession number: GCA_010093895.1) was used as an outgroup.

## Supplementary Information

**Additional file 1: Table S1.** Gene annotation in predicted transcripts of *Zalaria* sp. Him3.

## Data Availability

The datasets generated and/or analyzed during the current study are available in the DNA data bank of Japan (DDBJ, Shizuoka, Japan) repository. The accession numbers are: BPUN01000001–BPUN01000207 and the BioProject accession PRJDB12057.
